# A simplified up-down method (SUDO) for measuring mechanical nociception in rodents using von Frey filaments

**DOI:** 10.1186/1744-8069-10-26

**Published:** 2014-04-16

**Authors:** Robert P Bonin, Cyril Bories, Yves De Koninck

**Affiliations:** 1 Unité de neurosciences cellulaires et moléculaire, Centre de recherche de l’Institut universitaire en santé mentale de Québec, 2601 Chemin de la Canardière, Québec, QC G1J 2G3, Canada; 2 Department of Psychiatry and Neuroscience, Université Laval, Québec, Canada

**Keywords:** Mechanosensitivity, Pain measurement, Rodents, Allodynia, Nociception

## Abstract

**Background:**

The measurement of mechanosensitivity is a key method for the study of pain in animal models. This is often accomplished with the use of von Frey filaments in an up-down testing paradigm. The up-down method described by Chaplan et al. (J Neurosci Methods 53:55–63, 1994) for mechanosensitivity testing in rodents remains one of the most widely used methods for measuring pain in animals. However, this method results in animals receiving a varying number of stimuli, which may lead to animals in different groups receiving different testing experiences that influences their later responses. To standardize the measurement of mechanosensitivity we developed a simplified up-down method (SUDO) for estimating paw withdrawal threshold (PWT) with von Frey filaments that uses a constant number of five stimuli per test. We further refined the PWT calculation to allow the estimation of PWT directly from the behavioral response to the fifth stimulus, omitting the need for look-up tables.

**Results:**

The PWT estimates derived using SUDO strongly correlated (r > 0.96) with the PWT estimates determined with the conventional up-down method of Chaplan et al., and this correlation remained very strong across different levels of tester experience, different experimental conditions, and in tests from both mice and rats. The two testing methods also produced similar PWT estimates in prospective behavioral tests of mice at baseline and after induction of hyperalgesia by intraplantar capsaicin or complete Freund’s adjuvant.

**Conclusion:**

SUDO thus offers an accurate, fast and user-friendly replacement for the widely used up-down method of Chaplan et al.

## Background

An exaggerated nocifensive response to mechanical stimuli is considered a key indicator of abnormal sensory processing in most rodent models of pathological pain [1,2]. Mechanosensitivity can be determined as the threshold amount of force required to elicit a behavioral response, such as the withdrawal of a paw from the applied stimulus [3,4]. There are different methods available to estimate paw withdrawal threshold (PWT) with the use of von Frey filaments, but the up-down method of Dixon [5] as applied to rodents by Chaplan et al. [3] remains one of the most commonly used. Indeed, a survey of recent studies indicated that approximately 60% of publications where PWT was measured used thisIn the up-down method up-down method or a modified version of it [6].

**Figure 1:**
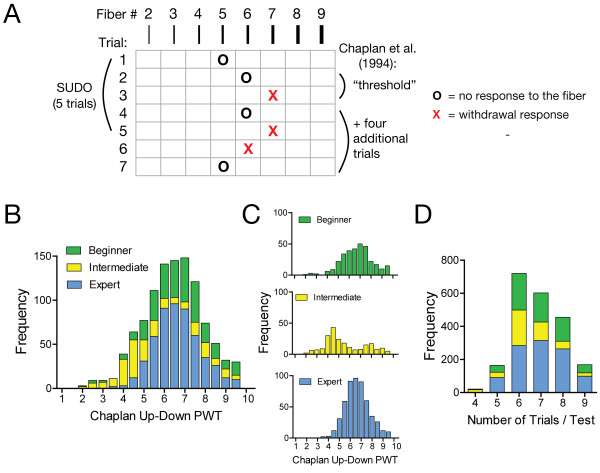
**Mouse mechanosensitivity measured with von Frey filaments. (A)** Illustration of the up-down von Frey testing paradigms described by Chaplan et al. [3] and the simplified up-down method (SUDO). **(B)** Frequency distribution of the paw withdrawal threshold (PWT) estimates calculated using the method of Chaplan et al., [3] for the mouse dataset that was analyzed (n = 1065). **(C)** Frequency distribution of the mouse PWT divided by experimenter to reveal data heterogeneity (beginner n = 318, intermediate n = 220, expert n = 527). **(D)** Frequency distribution of the total number of von Frey presentations (trials) per test.

In up-down methods of testing, a lack of response to a filament dictates that the next higher filament is used in the following stimulation, while a positive response dictates the use of the next lower filament. In the up-down method described by Chaplan et al. [3], the number of von Frey presentations in each trial is ultimately determined by the number of filament presentations required to approach the PWT. In this case, the PWT is assumed to exist in the vicinity of a stimulus level where the animal first changes its response pattern: a negative response followed by a positive response or vice versa (Figure 1A). Once this threshold is approached, another four von Frey presentations are done according to up-down rules and the PWT is calculated by combining the value of the final von Frey filament used with an adjustment factor based on the response pattern of the animal [3].

When testing is conducted with a conventional set of 8 different von Frey filaments [3], this method allows for a total of four to nine stimuli in each trial. This variability can become a source of bias since repeated testing can change the responsiveness of the animal. Such a change in responsiveness was described by Chaplan et al. [3], when prolonged testing sessions altered the PWT of normal, un-operated rats. Additionally, the need to keep track of variable numbers of stimuli can become cumbersome and error-prone when large groups of animals are tested simultaneously.

To alleviate these drawbacks, we developed a *S*implified *U*p-*Do*wn method (SUDO) for mechanosensitivity testing with von Frey filaments that uses a consistent number of stimuli without affecting PWT estimation. We propose using only five von Frey filament presentations per test; the minimum possible number when testing with a set of 8 filaments in an up-down manner. We implemented a further simplification for the estimation of PWT that employs a constant adjustment factor based solely on the response to the fifth stimulus, omitting the need for look-up tables for PWT calculation [3,5]. Using the SUDO method, we reanalyzed mechanosensitivity tests that were originally conducted with mice and rats using the method of Chaplan et al. [3]. We determined that SUDO effectively reproduces the PWT estimates calculated with the method of Chaplan et al. [3]. By reducing the number of stimuli to five in each trial we anticipate that SUDO will require, on average, nearly 30% fewer filament presentations and streamline testing and PWT estimation. Overall, we believe that the improvements offered by SUDO will improve mechanosensitivity testing times and accuracy, particularly when large groups of mice are used, and reduce training time for new users.

## Results

We first reanalyzed the results from 1065 previous mechanosensitivity measurements in C57BL/6 mice. The original measurements were conducted by three different experimenters following the method described by Chaplan et al. [3] (Figure 1A, B). Each of the three experimenters had different levels of experience with mechanosensitivity measurements using von Frey filaments and were classified as “beginner” (less than 3 months experience, n = 318), “intermediate” (more than 3 months experience, n = 220), and “expert” (more than one year experience, n = 527; Figure 1C). The data from the three experimenters were obtained from mice at baseline or with varying degrees of experimental hyperalgesia induced though several means that differed across experimenters. The population of PWT estimates in the intermediate data set calculated with the method of Chaplan et al. [3] were significantly different from the beginner and expert data sets (Kruskal-Wallis statistic = 83.8, P < 0.001; intermediate vs beginner: P < 0.001; intermediate vs expert: P < 0.001; beginner vs expert: P > 0.05). However, no further stratification by hyperalgesic condition was done to preserve the size and heterogeneity of the data sets. The average number of stimuli presented across all mouse trials was 6.8 (95% CI: 6.8 – 6.9; Figure 1D).

**Figure 2:**
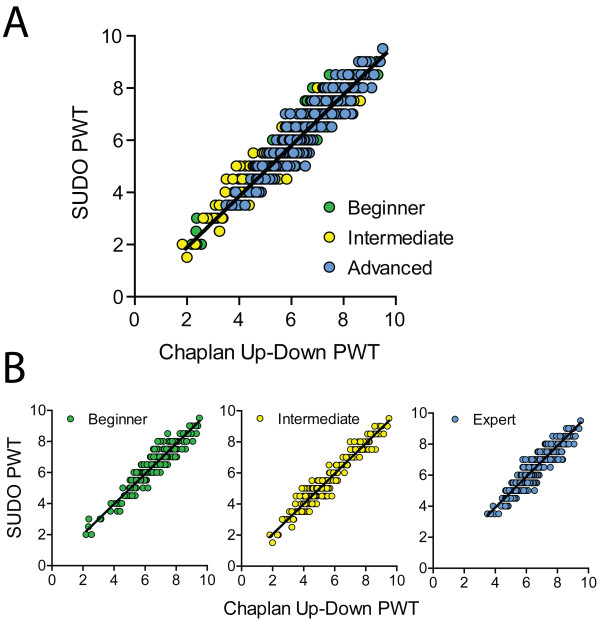
**Strong correlation between mouse paw withdrawal threshold (PWT) estimates calculated using the method of Chaplan et al. and SUDO. (A)** Correlation between PWT estimates determined using the two methods for the mouse dataset. **(B)** Correlation between PWT estimates from the two methods divided by experimenter.

The PWT estimates were reanalyzed with SUDO by taking the value of the fifth filament used in each testing sequence and adding or subtracting a value of 0.5 filament intervals if the response to the fifth filament was negative or positive, respectively (Figure 1A). When the entire mouse data set was tested without stratification by experimenter, SUDO produced PWT estimates that correlated extremely well with the estimates derived from the method of Dixon [5] (Pearson r = 0.96, P < 0.0001; Figure 2A). This correlation remained very high regardless if the original experimenter was a beginner (r = 0.96, P < 0.0001), intermediate (r = 0.97; P < 0.0001), or expert (r = 0.94; P < 0.0001; Figure 2B).

**Figure 3:**
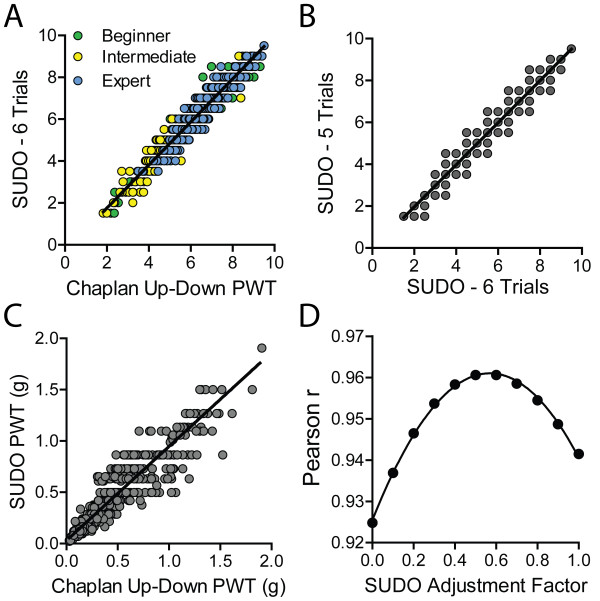
**Validation of the parameters of SUDO. (A)** Strong correlation between mouse paw withdrawal threshold (PWT) estimates calculated using SUDO with 6 von Frey presentations (trials) per test and the method of Chaplan et al. [3]. **(B)** Correlation between PWT estimates calculated with SUDO using five or six von Frey presentations. **(C)** Correlation between mouse PWT estimates when calculated as filament bending force using the up-down method of Chaplan et al. and SUDO with 5 von Frey presentations. **(D)** Effect of changing the adjustment factor for the PWT estimation for SUDO, demonstrating a peak correlation between PWTs calculated using the simplified method and the method of Chaplan et al. [3] with an adjustment factor near 0.5.

We further explored whether this correlation could be improved by using the value of the sixth filament used instead of the fifth filament. In this case the correlation remained very strong (r = 0.97; P < 0.0001; Figure 3A), although this correlation was not appreciably stronger than the correlation obtained through comparison with estimates from the fifth filament. Additionally, the PWT estimates derived from the fifth and sixth filament used were also very highly correlated (r = 0.96; P < 0.0001; Figure 3B), suggesting that there is no measureable benefit to increasing the number of filament presentations from 5 to 6. Additionally, we confirmed that the correlation of PWT estimates obtained with the two methods is still present after conversion from filament number to bending force. After conversion of both PWT estimates to force, the correlation was again very strong (r = 0.95, P < 0.0001; Figure 3C).

We next questioned whether the use of an adjustment factor of ± 0.5 stimulus intervals based on the response to the fifth filament was optimal. To test this, the adjustment factor was varied from 0 to 1 stimulus intervals in increments of 0.1, and was added or subtracted from the value of the fifth filament as previously done. These PWT estimates were correlated with the estimates from the method of Chaplan et al. [3], and the Pearson r values of the correlations were plotted against the adjustment factor (Figure 3D). The plot of correlation strength versus adjustment factor was fit with a non-linear quadratic function (r^2^ = 1.00) that indicated a maximum degree of correlation (r = 0.96) with an adjustment factor of ± 0.57. This maximal r value is equal to the r value obtained with an adjustment factor of 0.5 stimulus intervals, indicating that 0.5 is an ideal, or very close to ideal, adjustment value for the PWT calculation.

**Figure 4:**
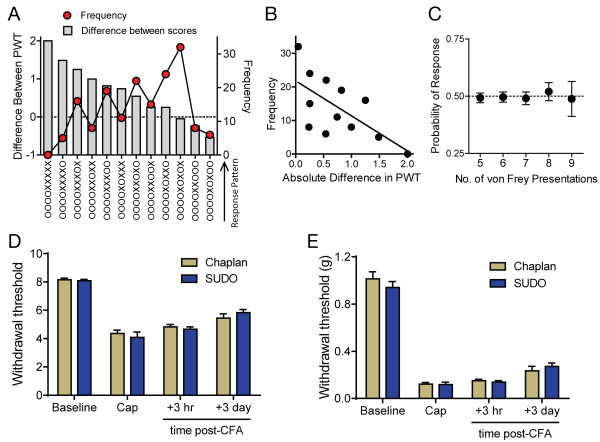
**Lack of sensitization within single mechanosensitivity tests in mice conducted using up-down methods. (A)** Observed frequency of each possible response pattern when 9 von Frey presentations were used in a test and the corresponding difference in the PWT estimates for each pattern as calculated using the method of Chaplan et al. [3] and SUDO. **(B)** Negative correlation between the magnitude of the difference in PWT estimates calculated using the two methods and the frequency with which each pattern occurred. **(C)** Probability of a positive nocifensive response by a mouse to the fifth through ninth von Frey filament presentation in all trials. Data in **(C)** are mean and 95% confidence interval. **(D)** PWT estimates measured in mice at baseline (n = 58), 3 hours after intraplantar injection of capsaicin (Cap; 5 μl, 0.5% w/v, n = 12), and 3 hours or 3 days after intraplantar injection of complete Freund’s adjuvant (CFA; 10 μl, n = 12) using the method of Chaplan et al. [3] and SUDO. The two testing methods were presented in a randomized crossover manner in each condition. PWT estimates were not significantly different between methods. **(E)** Same data as in **(D)** with PWT estimated expressed as force (g). PWT estimates were not significantly different between methods. Data in **(D)** and **(E)** are mean ± s.e.m.

The greatest difference between the PWT estimates obtained with the method of Chaplan et al. [3] or SUDO would occur in cases where a large change in response pattern occurs after the fifth filament used. For example, the pattern OOOOXXXXX would yield a withdrawal estimate of 6.5 using the scoring method of Dixon [5], but 8.5 when calculated with the simplified method. Because the largest differences are seen when the response pattern begins with four consecutive negative responses (“OOOO”) and 9 stimuli are presented per trial, we investigated all trials with 9 filament presentations (n = 166) to determine the frequency with which response patterns associated with large differences in scoring occurred (Figure 4A). We found a strong, negative correlation between the magnitude of the difference of the PWT estimates (SUDO vs. Chaplan et al.) for a particular response pattern and the frequency of occurrence of the pattern (r = −0.66, P = 0.019; Figure 4B). This suggests that large changes in mouse responding did not occur very frequently, which indicated that mice are unlikely to exhibit mechanical sensitization or desensitization throughout a single trial. To look at this possibility directly we calculated the probability that a mouse would respond to the fifth through the ninth filament presentation for each trial in the data set. We did not examine the first through fourth stimuli since the response probabilities in these trials will be largely determined by difference between the actual PWT and the starting stimulus level. We found that the response probability to any of the fifth through ninth presentation of filament in a single trial did not significantly differ from 0.5 (P > 0.05 for all comparisons), suggesting that mice neither sensitized nor desensitized in a single trial (Figure 4C).

We next prospectively determined whether SUDO and the method of Chaplan et al. [3] produce similar PWT estimates in mechanosensitivity tests. The PWT of mice was measured using both methods in a randomized cross-over manner at baseline (n = 58) and after intraplantar injection of capsaicin (n = 12) or complete Freund’s adjuvant (CFA; n = 12). We found no differences in the PWT estimates of mice measured with either method, either in terms of filament number (all P > 0.05; Figure 4D) or force (all P > 0.05; Figure 4E).

**Figure 5:**
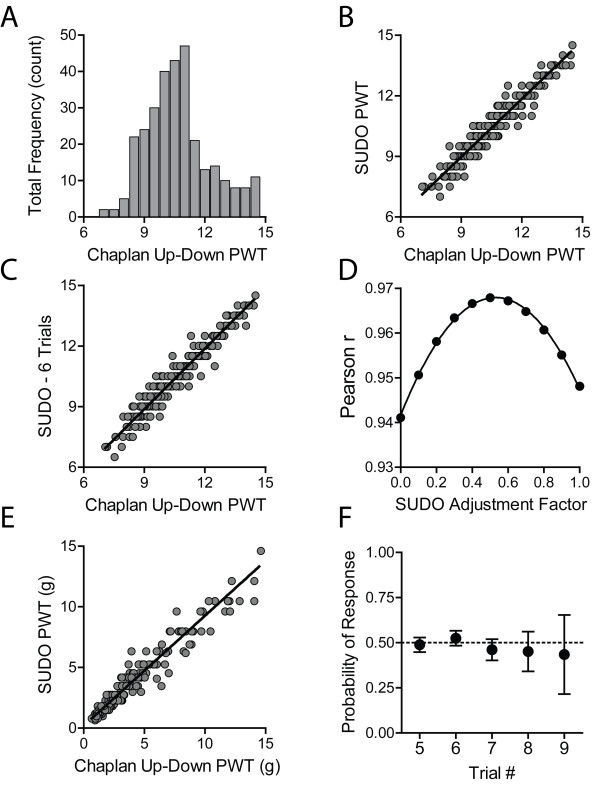
**SUDO is valid for PWT estimation in rats. (A)** Frequency distribution of the rat paw withdrawal threshold (PWT) estimates calculated using the method of Chaplan et al., ([3]; n = 300). **(B)** Strong correlation between PWT estimates determined using the method of Chaplan et al. [3] and SUDO. **(C)** Correlation between mouse PWT estimates calculated using six von Frey presentations (trials) and the method of Chaplan et al. [3]. **(D)** Effect of changing the adjustment factor in the simplified scoring method, demonstrating a peak correlation with an adjustment factor near 0.5. **(E)** Correlation between mouse PWT estimates calculated with the two methods when calculated as filament bending force. **(F)** Probability of a positive nocifensive response by a rat to the fifth through ninth von Frey filament presentation in all trials. Data in **(F)** are mean and 95% confidence interval.

To ascertain whether these modifications are also applicable to testing with other animals, we reanalyzed von Frey results from rats obtained following the method of Chaplan et al. [3]. 300 trials from a single experimenter were reanalyzed using SUDO (Figure 5A). The average number of stimuli presented in these trials was 6.6 (95% CI: 6.5 – 6.7). We again observed an extremely strong correlation between the PWT estimated obtained with the two methods (r = 0.98, P < 0.0001; Figure 5B). Similar to the reanalysis of the mouse data, reanalyzing the rat data using the value of the sixth filament used did not produce any further improvement of this correlation (r = 0.97; p < 0.0001; Figure 5C). This suggests that five filament presentations are sufficient to reproduce the PWT estimates for rats obtained with the method of Chaplan et al. [3]. We also tested whether an adjustment value of ± 0.5 stimulus intervals also produces the strongest correlation between PWT estimates from the two methods in the rat data set. The adjustment was again varied from 0 to 1 in increments of 0.1, and the plot of the Pearson r values and adjustment factor was fit with a non-linear quadratic function that had a maximum value of r = 0.98 at an adjustment value of 0.53 (Figure 5D). This indicates than an adjustment factor of 0.5 stimulus intervals is ideal, or close to ideal, for testing in rats. We also confirmed that the correlation between PWT estimated obtained with SUDO or the method of Chaplan et al. [3] did not change after converting the PWT estimates from fiber number to bending force (r = 0.98, P < 0.0001; Figure 5E). Similar to the mouse results, we also did not observe any indication of sensitization in the rat data: the response probability at the fifth through ninth filament presentation did not significantly differ from 0.5 (P > 0.05 for all; Figure 5F).

## Discussion

**Figure 6:**
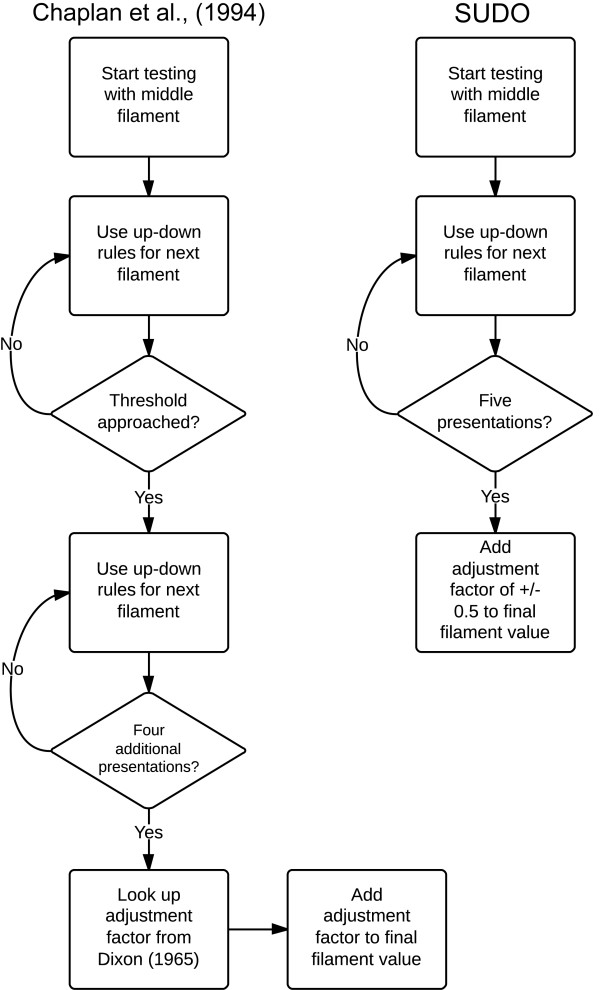
**Flowchart summarizing mechanosensitivity testing protocols of Chaplan et al. and SUDO.** In the method of Chaplan et al. [3], the threshold is considered to have been reached the first time that the response of the animal to a von Frey filament is different from the previous trial, after which four additional von Frey presentations are conducted in an up-down manner. At the end of the trial the paw withdrawal estimate (PWT) is calculated by taking the value of the final filament used and adding an adjustment factor derived from the response pattern using the calculations of Dixon [5]. In SUDO, only five von Frey filament presentations are done in each test and the PWT is estimated directly from the response to the final filament by incorporating a fixed adjustment factor based on the response to the fifth filament. See text for additional details.

Our analysis confirms that SUDO provides a reliable alternative to the up-down method of Dixon [5] as adapted by Chaplan et al. [3] for the estimation of PWT (Figure 6). We validated the use of five filament presentations in each test and the use a constant adjustment factor of 0.5 stimulus intervals for the simplified PWT estimate. There was no improvement in the PWT estimates obtained with six filaments as compared to five, which is the minimum possible in a set of eight or nine filaments when starting testing in the middle of the rage, indicating that the use of five von Frey presentations is sufficient for testing. Additionally, an adjustment factor of 0.5 stimulus intervals was found to be an ideal value in terms of conceptual simplicity and strength of correlation between the two methods.

No effort was made to distinguish between the different experimental populations in the data sets, where mechanical hypersensitivity was induced through different means. By grouping all the results together we created a much more heterogeneous data set than would be produced if the data sets were stratified by condition. We believe that this heterogeneous dataset is representative of data from mechanosensitivity experiments, where both normal and pathological conditions may be tested simultaneously and where the experimenter should to be blind to the particular experimental condition of the animal. Notably, the strongest correlation between PWT estimates was seen in datasets that covered the widest range of withdrawal threshold estimates (rat and “intermediate” set in mouse) indicative of the broadest range of observed mechanosensitivity. This implies that SUDO replicates the estimates obtained with the method of Chaplan et al. [3] irrespective of pathology or range of hyperalgesia. Our data from behavioral experiments support this, where the use of SUDO or the method of Chaplan et al. [3] produced similar PWT estimates in animals under baseline conditions or after the induction of hyperalgesia by intraplantar capaicin or CFA. Moreover, very strong correlations between scoring methods were obtained in datasets that were significantly different from each other (mouse intermediate vs. expert or beginner), demonstrating broad applicability of our method.

The simplifications proposed here are limited to tests that use a set of eight or nine filaments. Thus, SUDO will likely be applicable in any testing system where the relevant up-down testing range can be divided into eight or nine equally spaced stimulus levels. We did not test whether similar modifications can be made in systems with more than nine filaments, which would require a minimum of 6 or more stimuli presentations to cover the full testing range. However, increasing the minimum number of trials necessary diminishes the time savings from these simplifications and may exacerbate the possible testing bias created by repeated resting [3]. Additionally, it is unlikely that dividing the relevant sensory range into more than eight or nine stimuli levels will significantly enhance the accuracy of the PWT estimate given the relative insensitivity of the up-down method of Dixon to the spacing of the stimulus levels [5].

We saw no evidence of sensitization or changes in responsiveness over the single trials analyzed here. Indeed, the probability of a positive response to the fifth through ninth stimulus in a testing sequence was never significantly different from 0.5 in rats or mice. However, this finding does not exclude the possibility that sensitization can occur across trials during repeated testing, as reported by Chaplan et al. [3]. Because fewer stimuli are required for the simplified testing method here compared to the up-down method of Chaplan et al. [3], we speculate our approach will further reduce the possibility of sensitization occurring with repeated testing.

Our simplified method uses a fixed number of stimuli per trial that is independent from the response pattern of the mouse. This has several advantages. It ensures a constant number of stimuli per animal, which may help reduce measurement bias that could arise from large disparities in the number of stimuli given to different mice or rats. Additionally, using a constant number of stimuli is methodologically simpler than using a variable number of stimuli, which we believe would reduce training time for new experimenters and reduce the risk of experimenter error associated with the need to track variable stimulation numbers in large groups of animals. Finally, the use of only five stimuli equates to a time savings of nearly 30% compared to the average number of stimuli used in the data sets analyzed here. These time savings may become substantial when tests are conducted on large groups of animals and mice in particular, which typically move more and take longer to test than rats [4].

We further validate the use of a constant adjustment factor of 0.5 stimulus intervals to replace the adjustment factors calculated by Dixon [5]. The adjustment factors calculated by Dixon [5] are dependent on the response pattern throughout the trial and are derived using a maximum probability estimate to obtain the 50% response threshold. While this is a sound statistical approach for the estimation of the 50% response threshold, this degree of precision may not be necessary for the estimation of PWT as demonstrated by the very strong correlation between the PWT estimates derived using SUDO and the method of Chaplan et al. [3]. An adjustment factor of 0.5 stimulus intervals has the additional benefit of being a conceptually simple value, allowing for the quick manual addition or subtraction of half a stimulus interval to calculate the PWT estimate.

## Conclusion

The present study validates the use of SUDO for mechanosensitivity testing in rodents with von Frey fibers. Our simplified method effectively reproduces the PWT estimates obtained with the widely used method of Chaplan et al. [3], but with a faster and more user-friendly approach.

## Methods

### Data analysis

Data from previous von Frey tests in 1065 mice and 300 rats were entered into a spreadsheet for analysis. In each test, two separate trials were conducted and averaged to obtain the PWT. Tests were excluded if any of response patterns recorded did not correspond to the testing method described by Chaplan et al. [3] or would have required the use of filaments outside the specified range. The values of the von Frey filaments reported here represent the number of the filament within a complete set of 20 von Frey filaments that spans a range of force from 0.008 g to 300 g (Stoelting, Dale Wood, IL, USA). In the original mouse tests, filaments numbered 2 through 9 were used, while in rat tests filaments 7 through 14 were used. Testing always began with filament 5 for mice and filament 10 for rats, and the testing sequence progressed following an up-down sequence such that a positive response to a particular filament indicated the next lower value filament be used in the subsequent test, while a negative response indicated the next higher value filament be used [3]. Testing was stopped if a positive response to the lowest possible filament or a negative response to the highest possible filament was observed.

The PWT estimate was first calculated from the response pattern using methods described in Chaplan et al. [3]. For this, an adjustment factor was determined by the response pattern in the trial and added to the final filament value used in the trial (Figure 1A). The adjustment factors were obtained from the look-up tables developed by Dixon for this purpose [5]. For SUDO, the PWT estimate was calculated by taking the value of the fifth filament used in each test and adding an adjustment value of ± 0.5 stimulus intervals (Figure 1A). The adjustment factor was positive if there was no response to the fifth filament of the sequence to generate a PWT slightly higher than the fifth filament value, or negative if there was a withdrawal to generate a PWT slightly lower than the fifth filament value. Because whole numbers were used for the filament values, this adjustment corresponded to an actual adjustment of ± 0.5 from the value of the fifth filament used. In some instances a variant of SUDO was used in which PWT was derived from the sixth filament used in the sequence, or the absolute adjustment factor was varied from 0 to 1 in increments of 0.1 stimulus intervals.

The use of whole numbers to represent the filament value is a valid approach since the estimation of the PWT using the up-down method of Dixon [5] assumes that the stimuli are equally spaced on an appropriate scale. It would also have been appropriate to use the manufacturer-provided filament values (e.g., 2.83, 3.22, 3.61, etc.), which are logarithmically related to the actual bending force of the filament and can also be fitted with a linear relation for the calculation of the 50% withdrawal thresholds. However, we preferred the use of whole numbers because they allowed for a much simpler calculation of PWT by using an adjustment factor of ± 0.5 based on the stimulus intervals, yet do not alter the final outcome of the experiment. Moreover, it is equally possible to calculate the bending force of the filament from either the filament number of manufacturer-provided filament numbers.

Notably, different methods of inducing experimental pain hypersensitivity were used in the original experiments by the different experimenters, including intraplantar injection of capsaicin (mice; beginner, intermediate) or CFA (mice; intermediate), experimental autoimmune encephalomyelitis (mice; expert), repetitive paw stimulation (mice; beginner), and nerve injury (rats). We did not distinguish between naïve or hyperalgesic animals in any of the original data sets since the degree of mechanical hypersensitivity can vary greatly both over time and between different experimental conditions, requiring the creation of many datasets for comparison with small sample sizes. We further reasoned that the heterogeneity in mechanosensitivity produced by pooling all experimental conditions is more representative of the variability that would often be observed in experiments designed to study mechanical hypersensitivity.

In some cases the PWT was converted from filament number to force, and expressed in grams using the equation:

$${\mathrm{PWT}}_{\mathrm{force}}=10^{\left(x*F+B\right)},$$

where F is the PWT calculated in terms of filament number using either SUDO or the method of Chaplan et al. [3]. x and B were determined from a linear regression of the logarithm of the empirically measured filament bending force plotted against the filament number using the equation:

$$\mathrm{Log}\left(\mathrm{bending}\;\mathrm{force}\right)=x*\mathrm{Filament}\;\mathrm{number}+B.$$

Because different filament sets were used for mice and rats, the linear regression for each set produced different constants. For the mouse filament set, x = 0.240 and B = −2.00, and for the rat filament set x = 0.182 and B = −1.47. The two trials in each test were converted to force before averaging to obtain the PWT in terms of filament bending force.

Statistical analyses were performed using Prism 5 (GraphPad, San Diego, CA, USA). Linear regression, correlation, or non-linear curve fitting were used as appropriate. Mouse data sets for beginner, intermediate and expert were compared using a Kruskal-Wallis test followed by Dunn’s multiple comparison post-test to compare between groups. To analyze the response probabilities, the responses at each filament presentation were compared to theoretical a value of 0.5 using a Wilcoxon signed rank test.

### Mechanosensitivity testing

All prospective mechanosensitivity experiments with animals were conducted in accordance with the guidelines from the Canadian Council on Animal Care and approval of the Laval University Animal Care Committee. PWT was measured in adult male C57BL/6 mice using von Frey filaments 2 through 9. Mice were placed in acrylic chambers (5.5 × 10 cm) suspended above a wire mesh grid and allowed to acclimatize to the testing apparatus for 1 hour prior to experiments. When the mouse was not moving the von Frey filaments were pressed against the plantar surface of the paw until the filament buckled and held for a maximum of 3 seconds. A positive response was noted if the paw was sharply withdrawn on application of the filament. Flinching immediately upon removal of the filament was also considered a positive response as previously described [3]. Testing began with filament number 5 and progressed according to an up-down method. Mice were randomly assigned to be tested either with the method of Chaplan et al. [3] or SUDO. After the first measurement of PWT, a second measurement of PWT was conducted using the alternate test. In some prospective experiments, hyperalgesia was induced by intraplantar injection of capsaicin (0.5% w/v, 5 μl) or complete Freund’s adjuvant (CFA; 10 μl) in one hind paw under brief anesthesia with isoflurane (<3 minutes, 4% isoflurane). PWT was measured 3 hours after intraplantar injection of either compound and again 3 days after injection of CFA. The PWT estimates derived using SUDO or the method of Chaplan et al. [3] for each condition were compared using a paired *t*-test in Graphpad Prism 5.

## Abbreviations

CFA: Complete Freund’s adjuvant; PWT: Paw withdrawal threshold; SUDO: Simplified up-down method.

## Competing interests

The authors declare that they have no competing interests.

## Authors’ contributions

RPB conducted the behavioral experiments and analyzed the data, and all authors designed the study and wrote the manuscript. All authors read and approved the final manuscript.
